# Retrospective content analysis of consumer product reviews related to chronic pain

**DOI:** 10.3389/fdgth.2023.958338

**Published:** 2023-04-24

**Authors:** Jungwei W. Fan, Wanjing Wang, Ming Huang, Hongfang Liu, W. Michael Hooten

**Affiliations:** ^1^ Department of Artificial Intelligence and Informatics, Mayo Clinic, Rochester, MN, United States; ^2^ Department of Computer and Information Science, University of Pennsylvania, Philadelphia, PA, United States; ^3^ Department of Anesthesiology and Perioperative Medicine, Mayo Clinic, Rochester, MN, United States

**Keywords:** chronic pain, data mining, online product reviews, consumer health informatics, retrospective study

## Abstract

Chronic pain (CP) lasts for more than 3 months, causing prolonged physical and mental burdens to patients. According to the US Centers for Disease Control and Prevention, CP contributes to more than 500 billion US dollars yearly in direct medical cost plus the associated productivity loss. CP is complex in etiology and can occur anywhere in the body, making it difficult to treat and manage. There is a pressing need for research to better summarize the common health issues faced by consumers living with CP and their experience in accessing over-the-counter analgesics or therapeutic devices. Modern online shopping platforms offer a broad array of opportunities for the secondary use of consumer-generated data in CP research. In this study, we performed an exploratory data mining study that analyzed CP-related Amazon product reviews. Our descriptive analyses characterized the review language, the reviewed products, the representative topics, and the network of comorbidities mentioned in the reviews. The results indicated that most of the reviews were concise yet rich in terms of representing the various health issues faced by people with CP. Despite the noise in the online reviews, we see potential in leveraging the data to capture certain consumer-reported outcomes or to identify shortcomings of the available products.

## 1. Introduction

Chronic pain (CP) lasts for over 3 months. CP is complex in etiology and can occur anywhere in the body, making it difficult to effectively treat and manage. CP is one of the most debilitating conditions that impose physical and mental burdens and incur significant socioeconomic costs (1). According to the US Centers for Disease Control and Prevention, CP contributes to an estimated $560 billion each year in direct medical cost plus the associated productivity loss. In 2016, approximately 20.4% (50 million) of US adults had CP, with 8.0% (19.6 million) experiencing high-impact CP that frequently restrains life or work activities (2). There is a pressing need for new research to summarize the common health issues and choices in CP management, through seeking not only formal medical treatments but also any self-sufficient approaches, such as using over-the-counter (OTC) products.

The market of OTC analgesics in the United States generated $5.2 billion in 2018, amounting to 24% of the overall OTC pharmaceutical revenue that year. The market is projected to grow to $5.9 billion in 2023 (3). Given the skyrocketing e-commerce fueled by the COVID-19 pandemic, it is ever critical to find efficient methods to understand the health issues faced by online consumers and how they address these issues through OTC products. Modern online shopping platforms offer an unprecedented opportunity for the secondary use of consumer-generated data in health informatics research. By comparing Amazon product reviews and clinical trial data, de Barra (4) showed that the reviews tended to be from consumers who experienced good outcomes and would therefore be biased. Maharana et al. (5) analyzed Amazon product reviews associated with the Food and Drug Administration safety recalls and developed machine learning methods to automate the detection of such unsafe products. Using the same Amazon review dataset as in this study, Torii et al. (6) estimated that 22% of the health-related reviews on grocery products were about adverse effects, and pain was the most frequently mentioned problem. As a constantly growing corpus, these massive customer reviews have become a valuable resource to gain first-hand insights into consumer experiences with CP management products, considering many analgesics are available online without requiring a prescription.

For the above rationales, we conducted an exploratory data mining study that analyzed CP-related Amazon product reviews. Our analyses characterized the review language, the reviewed products, the representative topics, and the network of comorbidities mentioned in the reviews. The multifaceted findings represent a descriptive overview of the health issues and contexts, the self-management approaches using the OTC products, and narrative snippets that reflect the experiences from using the products reported by consumers with CP.

## 2. Materials and methods

### 2.1. Data source and overview of analysis

The dataset consisted of publicly available Amazon product reviews posted between 2001 and 2014 (7). The source reference did not specify, but it is inferred to be a random subset of reviews retrieved from the Amazon US website and predominantly in English. We limited our analysis within the Health & Personal Care category (346,355 reviews) and filtered by including only those that contained “chronic pain” in the text (1,589 reviews). Within the filtered subset, we performed various descriptive analyses regarding the language properties, product categories, and disorders that co-occurred in the review texts and applied unsupervised topic detection for representative topics.

### 2.2. Descriptive analysis

JSON files of the reviews and product metadata were parsed to extract data fields including the product ID, consumer ID, review text, and the rating score (ranging from 1 to 5 stars). Sentences of the review texts were detected by applying the sentence chunker in the *stanza* natural language processing (NLP) library (8). On top of these preprocessed materials, the following analyses were performed:

1. Summary of the review language: We calculated descriptive statistics for the number of reviews by each consumer, the length of the reviews, and the reading level of the texts measured by the Dale–Chall readability score (9). These provide rough measures to gauge the complexity of the review texts as a standalone analysis itself and are not meant to be interpreted in association with any pain management product or behavior.
2. Summary of the reviewed products: We calculated the number of reviews per product and prevalence statistics based on the hierarchical categories that came with the product metadata. This gives an overview of the common product types purchased by consumers that they will probably use to manage their CP.
3. Summary of the mentioned disorders: The disorders mentioned in the review texts were identified by using *stanza*'s NCBI disorder extraction model (10), a deep-learning model named entity recognition (NER) modeltrained from the NCBI disease corpus (11). The disorders found in the same reviews were then fed as inputs into the *Gephi* network analysis program (12) to derive a co-occurrence network and compute properties such as node centrality. Betweenness centrality was computed to quantify the relative importance of each disorder node in terms of influencing the information flows in the network—it measures the number of times a node lies on the shortest paths between other pairs of nodes. The centrality weights were verified by the co-occurring frequencies of the disorder MeSH terms with chronic pain (MeSH ID: D059350), leveraging the 2022 MEDLINE co-occurrences file (13).We assessed whether the hub disorders in the network tend to be those commonly reported with CP in the literature. We also applied Blondel et al.'s community detection algorithm (14) to identify closely associated disorder nodes in the network. In *Gephi*'s implementation, the resolution coefficient regulates the community sizes; a lower resolution yields more (and smaller) communities. We empirically tuned the coefficient toward grouping more relevant disorders into the same communities.
4. Topic modeling of the review contents: Latent Dirichlet allocation (LDA) (15) is a widely used generative statistical approach for identifying topics, modeled through latent variables that are fit by intaking the observable words and documents from a corpus. Specifically, the *LDAvis* (16) program was used to identify the topics and their representative terms in the reviews. We set the relevance parameter *λ *= 0.6 as suggested by the program's reference and explored the number of topics from 5 to 20. We used bigrams as the term units because the unigrams tended to be vague due to splitting meaningful phrases.

### 2.3. Review rating analyses

To assess whether there was any disorder-specific consumer (dis)satisfaction (i.e., significantly higher or lower rating tendency), we performed *χ*^2^ tests on the rating distribution for disorders with a sufficient sample size (>30 reviews) against that of the entire review set. The null hypothesis is that the review ratings were independent of the disorder; under the hypothesis, the distribution of 1–5 stars in any specific disorder subset would be closely mirroring that of the entire study cohort.

To identify specific phrases representing those reviews with high vs. lower ratings, we applied text classification to reveal the discriminative features. The reviews rated 5 stars were used to represent the “high” class, and those with 1 or 2 stars were grouped into the “low” class. After removing stopwords, bigram and trigram features (for better interpretability than unigrams) were generated from the review texts. The features were weighted by the term frequency-inverse document frequency (TF-IDF) scheme, applying a minimum DF of 10. For the high vs. low rating, a logistic regression binary classifier (with L2 penalty and regularization term *C* = 0.1) was then trained on a random 70% split of the data and the remaining 30% was held for applying the SHapley Additive exPlanations (SHAP) program (17) to extract the important features that differentiate the two classes.

## 3. Results

### 3.1. Summary of the review language

**Figure 1:**
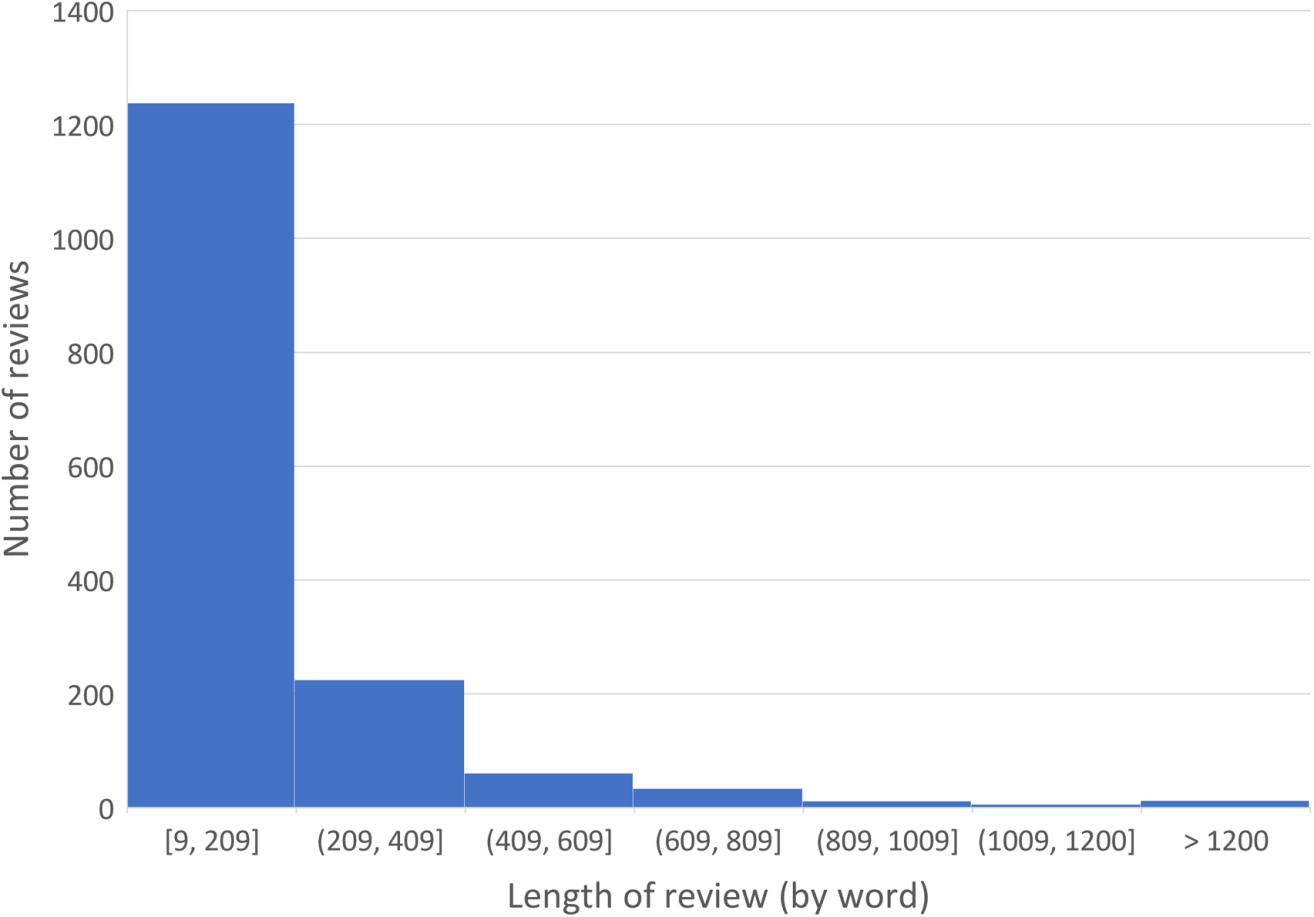
Distribution of the length of the reviews by the number of words.

**Figure 2:**
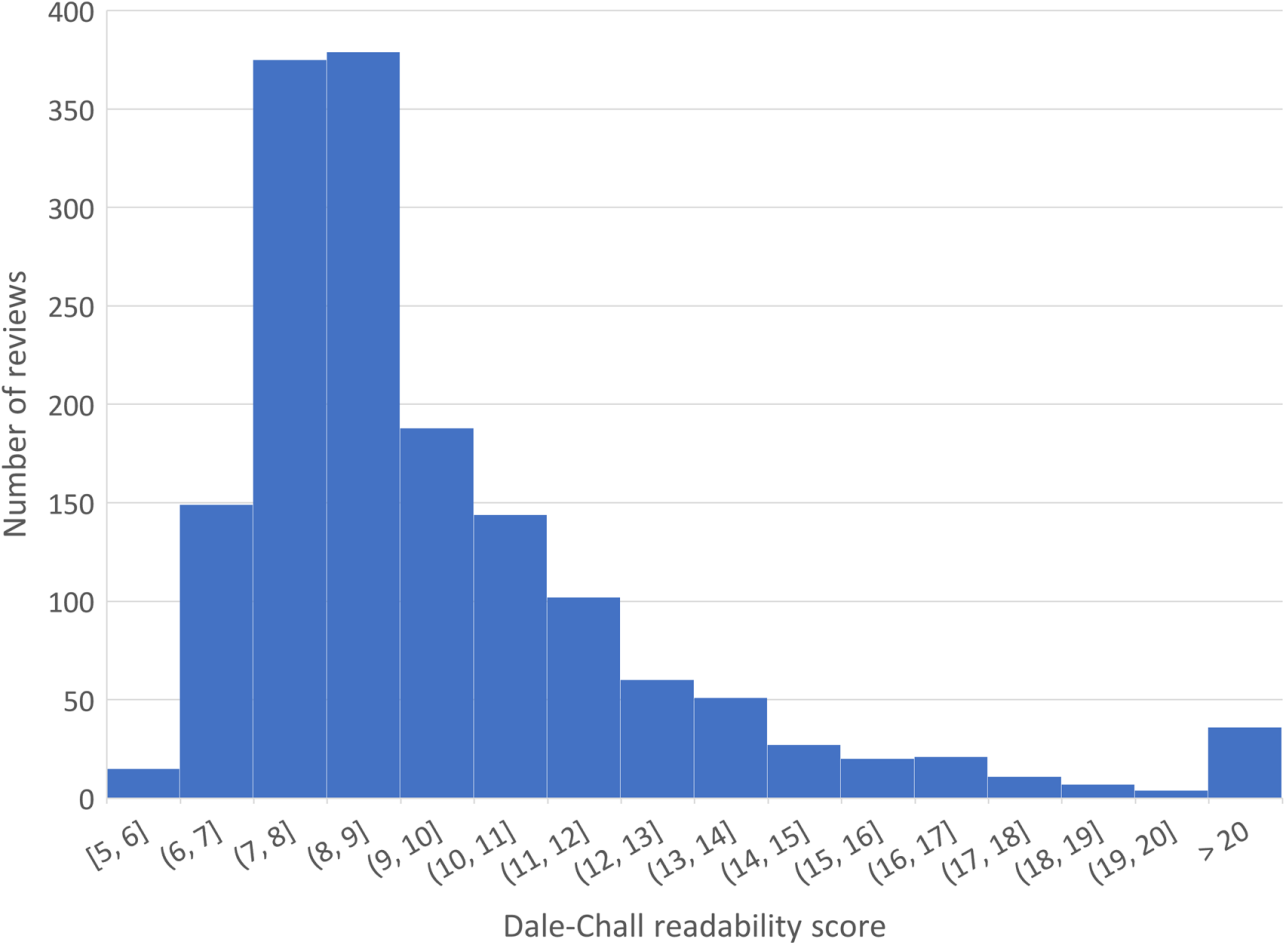
Distribution of the Dale–Chall readability score.

A total of 1,589 chronic pain-related reviews on 1,098 products were posted by 1,466 distinct consumers, of whom 1,382 posted only one review and the maximum was by one consumer who posted 14 reviews. The median length of the reviews was 107 words (min = 9, max = 2,392); see Figure 1 for the distribution of review length measured by the number of words. The review with 2,392 words contained excerpts of scientific articles used by the consumer to support their opinion. The reading level of the review texts, measured by the Dale–Chall readability score, is shown in Figure 2. We can see that the mass peaks around the score range of 7–9, which corresponds to the level that can be easily understood by a 9th–12th (high school) grader. An inspection into a few reviews with a score of >40 (i.e., difficult to read) found that they either contained many long sentences or contained many technical terms not used in general English (e.g., ECRB tendon, OTC NSAID, TENS Unit, and Naproxen).

### 3.2. Summary of the reviewed products

**Figure 3:**
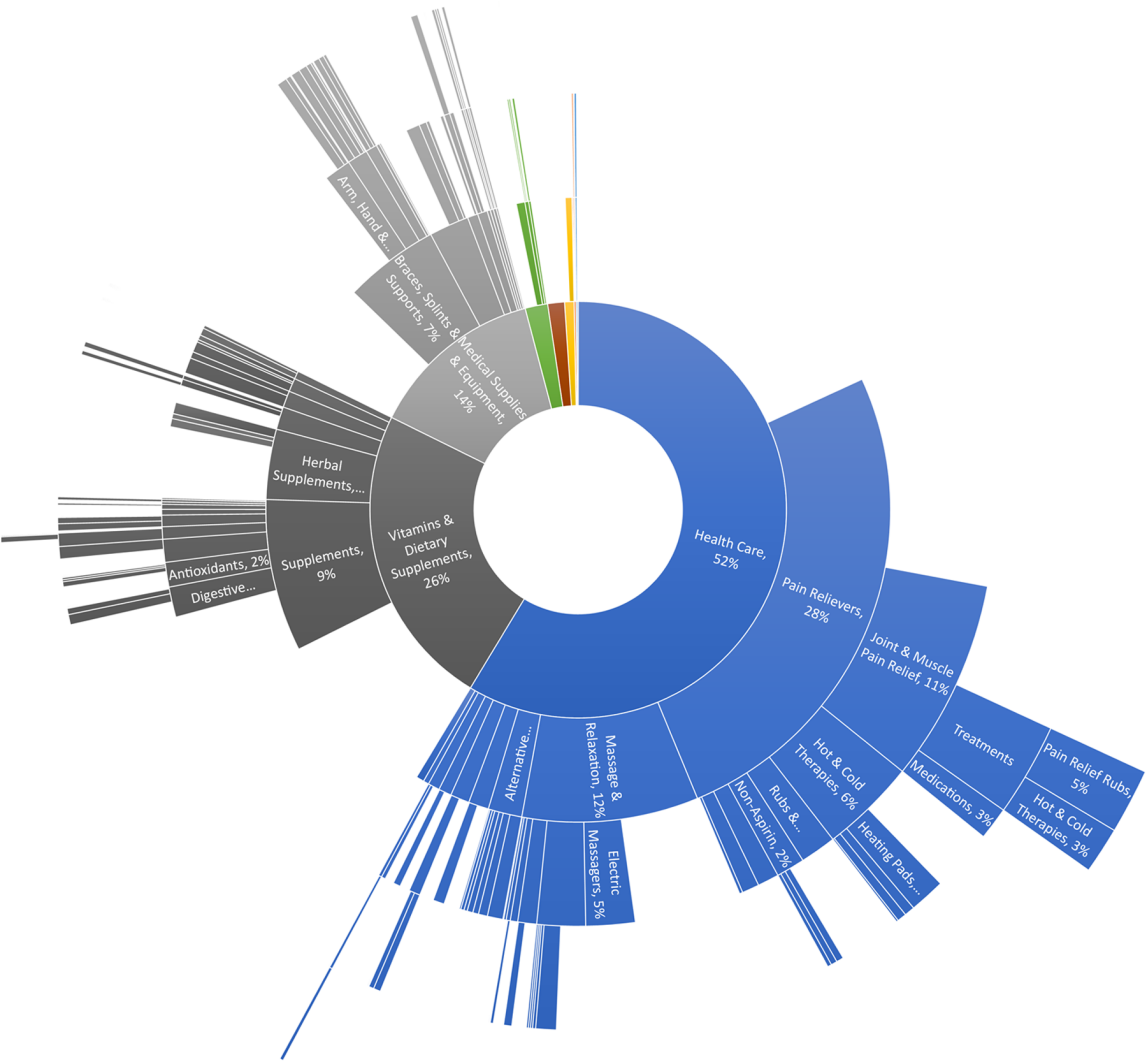
Sunburst chart of the product categories. These are based on 1,098 distinct products, with at least one review text mentioning chronic pain.

Among the 1,098 reviewed products, the majority (81.7%, *n* = 897) had only one review that mentioned CP. The product that received a maximum of 50 reviews was a topical anti-inflammatory cream. The hierarchy of the product categories is illustrated in the sunburst chart in Figure 3. Starting from the center, three major sections pan out:

1. Healthcare (52%) – This label serves more as a middle anchor layer that subsumes heterogeneous subcategories led by the following:
  a. Pain relievers (28%) – This covers oral pain medications (e.g., acetaminophen and ibuprofen), external analgesic ointment, and heat/cold therapy packs.
  b. Massagers and relaxants (12%) – This includes mainly external massage equipment (especially for back pain) and a minor proportion of aromatherapy items.
2. Vitamins and dietary supplements (26%) – This includes joint health products such as chondroitin and glucosamine. One herbal supplement that stood out was turmeric (curcumin). Magnesium led among the minerals. Other noticeable subcategories include antioxidants and digestive supplements.
3. Medical supplies and equipment (14%) – Compared to the massage equipment above, this category covers physical supporting braces, mobility aids, and physical therapy aids.

### 3.3. Summary of the mentioned disorders

**Figure 4:**
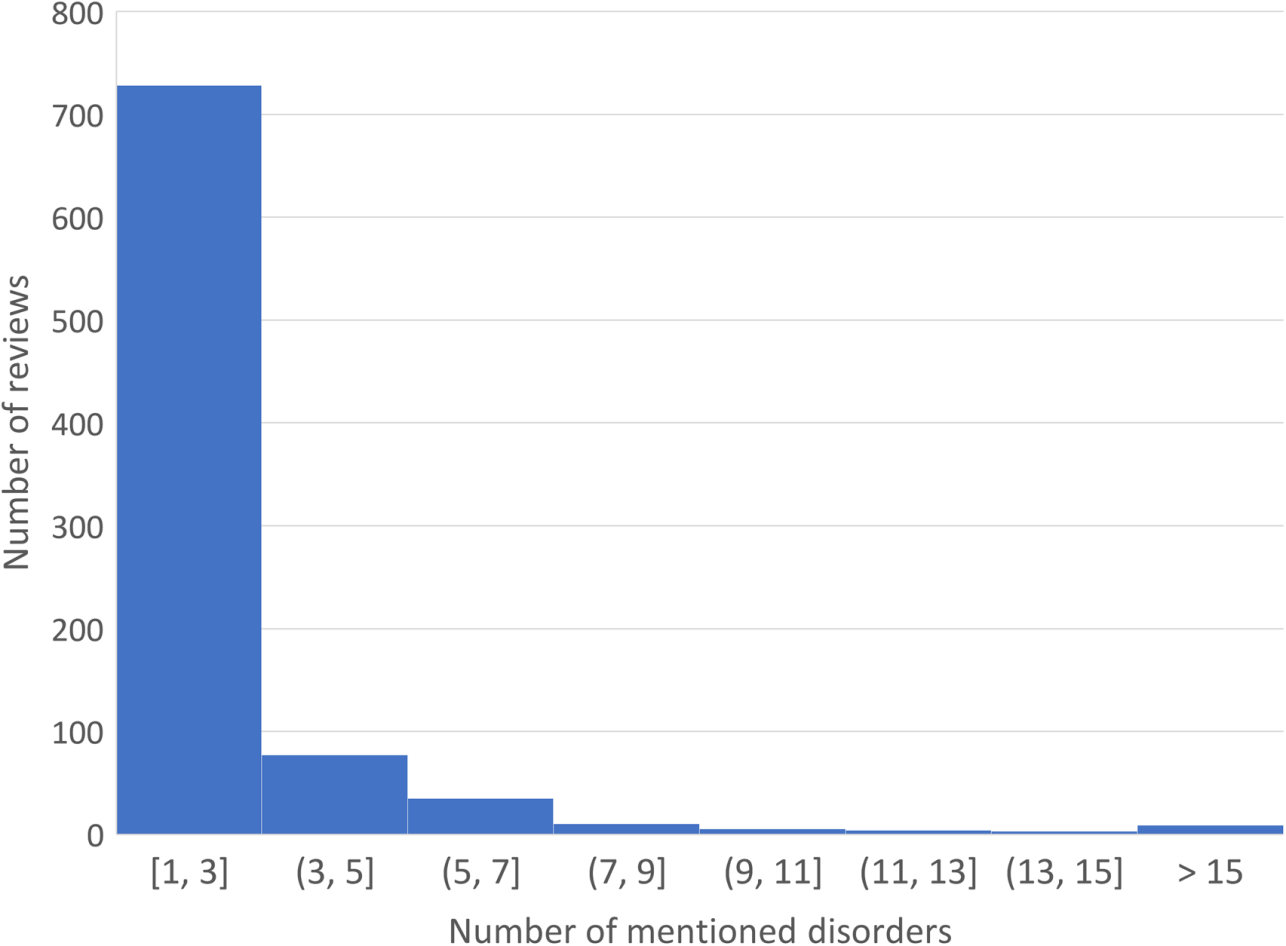
Distribution of the disorder terms mentioned per review.

**Figure 5:**
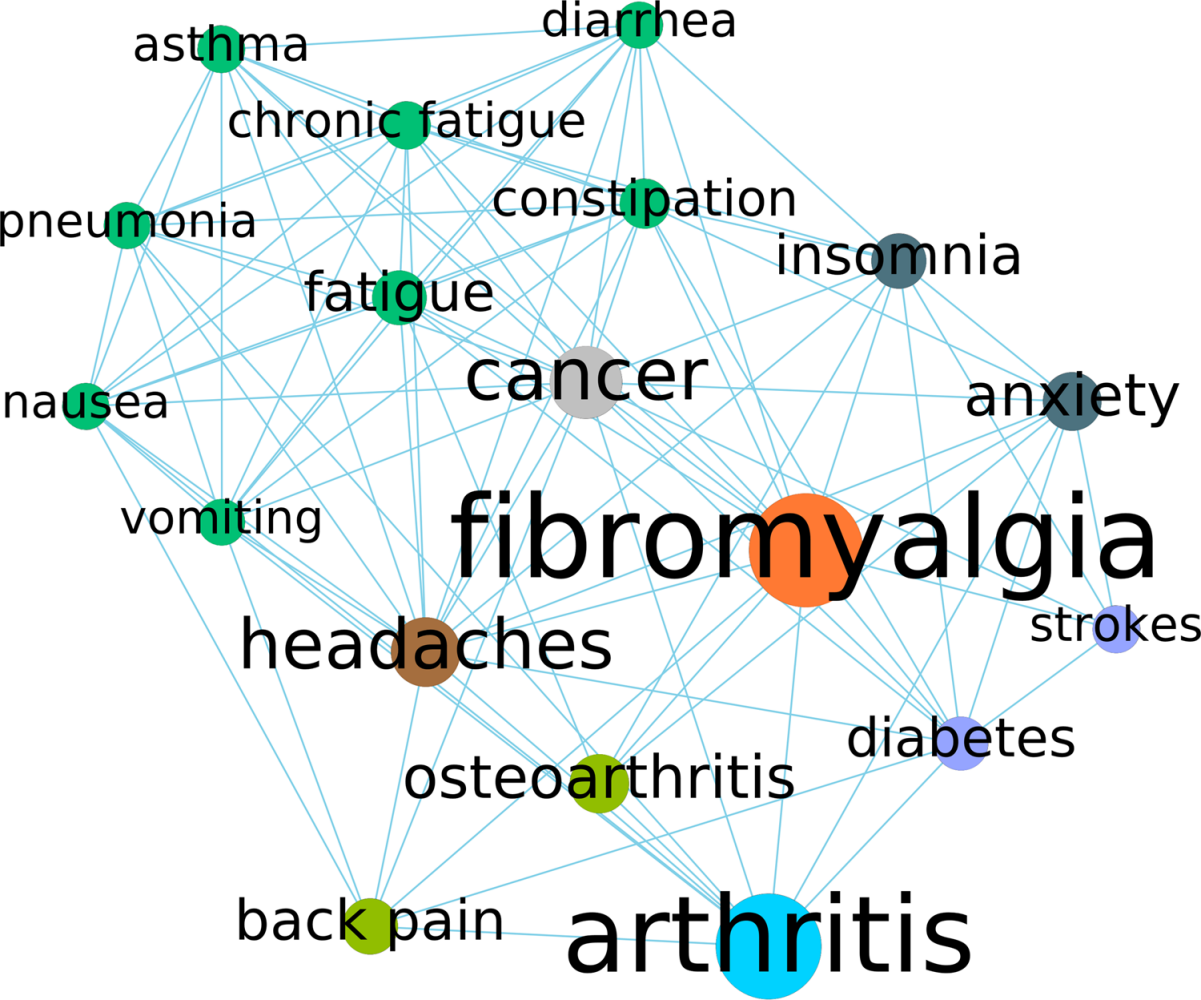
Network of the disorders with a node degree above 50, which was arbitrarily determined for readability without loss of representative information. The edges shown here are only those among the node that remained after the filtering. The node sizes are proportional to the betweenness centrality, and the node colors correspond to subgroups determined by Blondel et al.'s community detection algorithm with a resolution coefficient of 0.8.

Among the 1,589 reviews, 871 had at least one disorder term (other than CP) identified by the *stanza* biomedical NLP module. By manual review of 100 random disorder extractions, the NLP precision was estimated to be 90% (see Supplementary data). The distribution of the other identified disorders per review is shown in Figure 4. The majority mentioned 1–3 disorders, with the maximum mentioning 48 disorders in one review. Figure 5 shows the network analysis based on in-review co-occurrences of the disorders. Fibromyalgia and arthritis stand out as two leading hubs (by betweenness centrality) that have a strong influence on the CP disorder network. The node coloring by community detection also grouped comorbidities of coherent mechanism, e.g., cardiovascular (diabetes and stroke) or mental stress (anxiety and insomnia). As a simple validation of the hub nodes corresponding to published knowledge, Table 1 lists the disorders in descending order of their MeSH co-occurrence frequency with the MeSH term Chronic Pain in the 2022 MEDLINE co-occurrences file. We can see that the disorders frequently published in the CP literature tend to be the hubs in the disorder network constructed from consumer reviews. The two ends do not always align perfectly, though; e.g., back pain is not the largest hub in the network but tops the list among the disorders published about CP.

**Table 1:** Frequency and rank of the Figure 5 disorders as co-occurring MeSH terms with chronic pain (MeSH ID: D059350).

Disorder shown in the Figure 5 network derived from product reviews	MeSH ID	Number of times co-occurred with chronic pain in PubMed indices	Rank
Back pain^a^	D017116	331	9
Fibromyalgia	D005356	91	27
Cancer	D009369	30	87
Osteoarthritis	D010003	29	91
Fatigue	D005221	23	108
Insomnia	D007319	15	167
Headaches	D006261	9	273
Strokes	D020521	7	340
Constipation	D003248	7	363
Diabetes	D003920	6	384
Vomiting	D014839	3	701
Nausea	D009325	3	844
Chronic fatigue	D015673	3	948
Diarrhea	D003967	2	1,204
Pneumonia	D011014	1	5,144

This facilitates inspecting whether the major chronic pain-related disorders in the product reviews also co-occur frequently with chronic pain in the literature.

^a^ The concept of back pain (D001416) did not co-occur with chronic pain, so its hyponym low back pain (D017116) was used.

### 3.4. Findings from the review rating analyses

In the entire dataset, Table 2 shows that more than 86% of the reviews received a rating of 4 or 5 stars, indicating decent customer satisfaction overall. The *χ*^2^ tests for the two disorders mentioned by more than 30 reviews, fibromyalgia and arthritis, did not find significant differences in either of the disorder-specific rating distributions compared to the entire set.

**Table 2:** *Χ*^2^ tests for disorder-specific rating distribution against that of the entire dataset.

Review set (number of reviews)	Rating (number of stars)					*χ*^2^ statistic (*p*-value)
	1	2	3	4	5	
Full set (*n* = 1,589)						
Observed	63	57	85	216	1,168	–
Percentage	4.0	3.9	5.3	13.6	73.2	
Fibromyalgia (*n* = 127)						
Observed	3	5	4	13	102	3.85 (0.43)
Expected	5.0	4.6	6.8	17.3	93.4	
Arthritis (*n* = 107)						
Observed	2	2	5	12	86	3.20 (0.53)
Expected	4.2	3.8	5.7	14.5	78.7	

**Figure 6:**
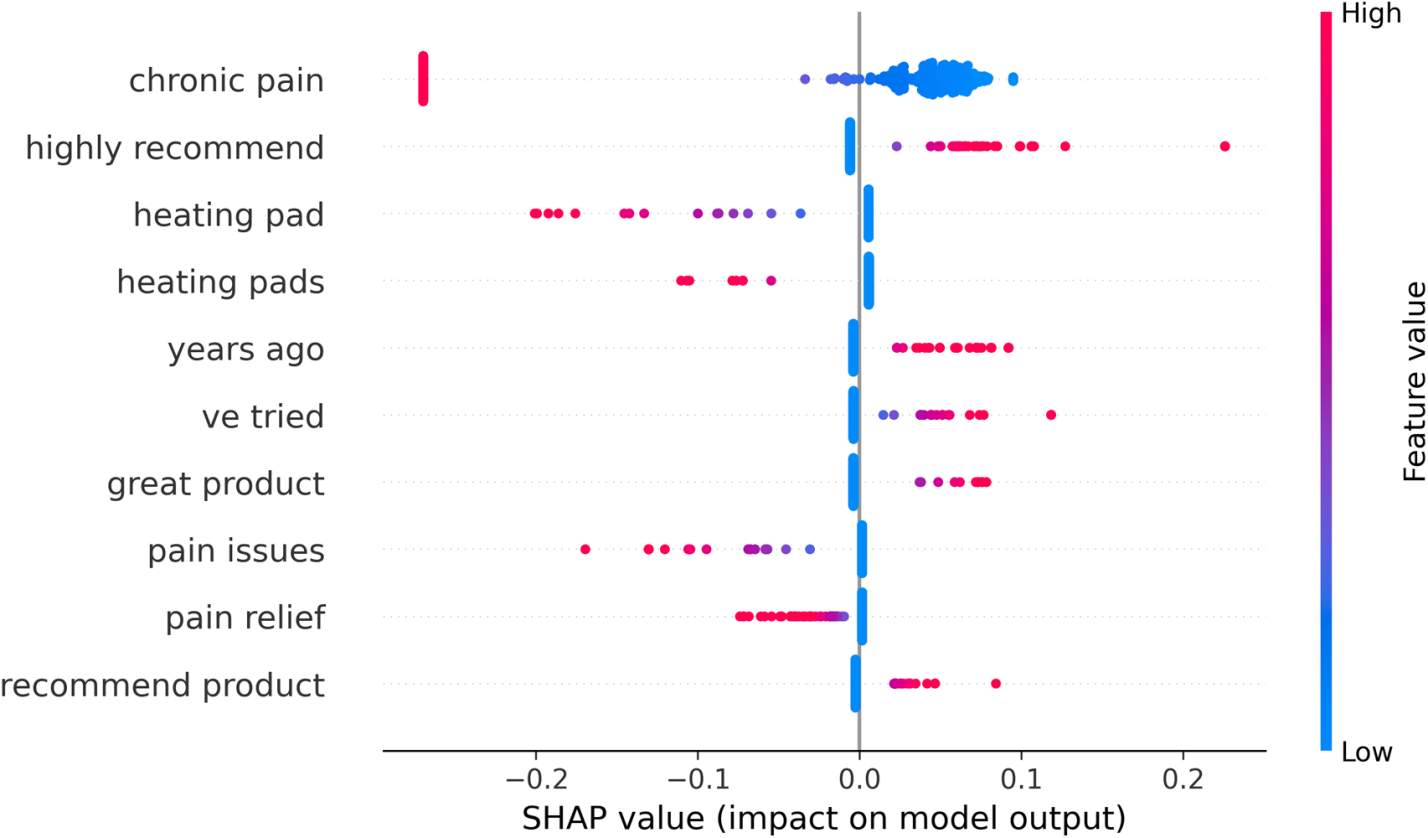
Shapley Additive exPlanations (SHAP) beeswarm plot showing the top 10 features in classifying the review texts of high (5 stars) vs. low rating (1 or 2 stars). Each data point represents a review text being classified. A positive larger SHAP value (the *x*-axis) indicates stronger support for a higher rating, and the opposite indicates support toward a lower rating. The color coding stands for the weight (hereby TF-IDF) assigned to the specific feature in the specific text. For example, “highly recommend” was a heavily weighted feature in many reviews that drove the classification toward the high rating end.

Figure 6 shows the top 10 features that differentiate the high vs. low rating reviews. Many reviews in which “chronic pain” has a low TF-IDF weight (blue) were associated with 5-star ratings, while several reviews with “chronic pain” of high TF-IDF weight (fuchsia) received negative reviews. Other features that are pro-negative rating included “heating pad(s),” “pain issues,” and “pain relief.” On the contrary, the pro-positive rating features were “highly recommend,” “years ago,” “ve tried,” “good product,” and “recommend product.”

### 3.5. Topic modeling of the review contents

**Figure 7:**
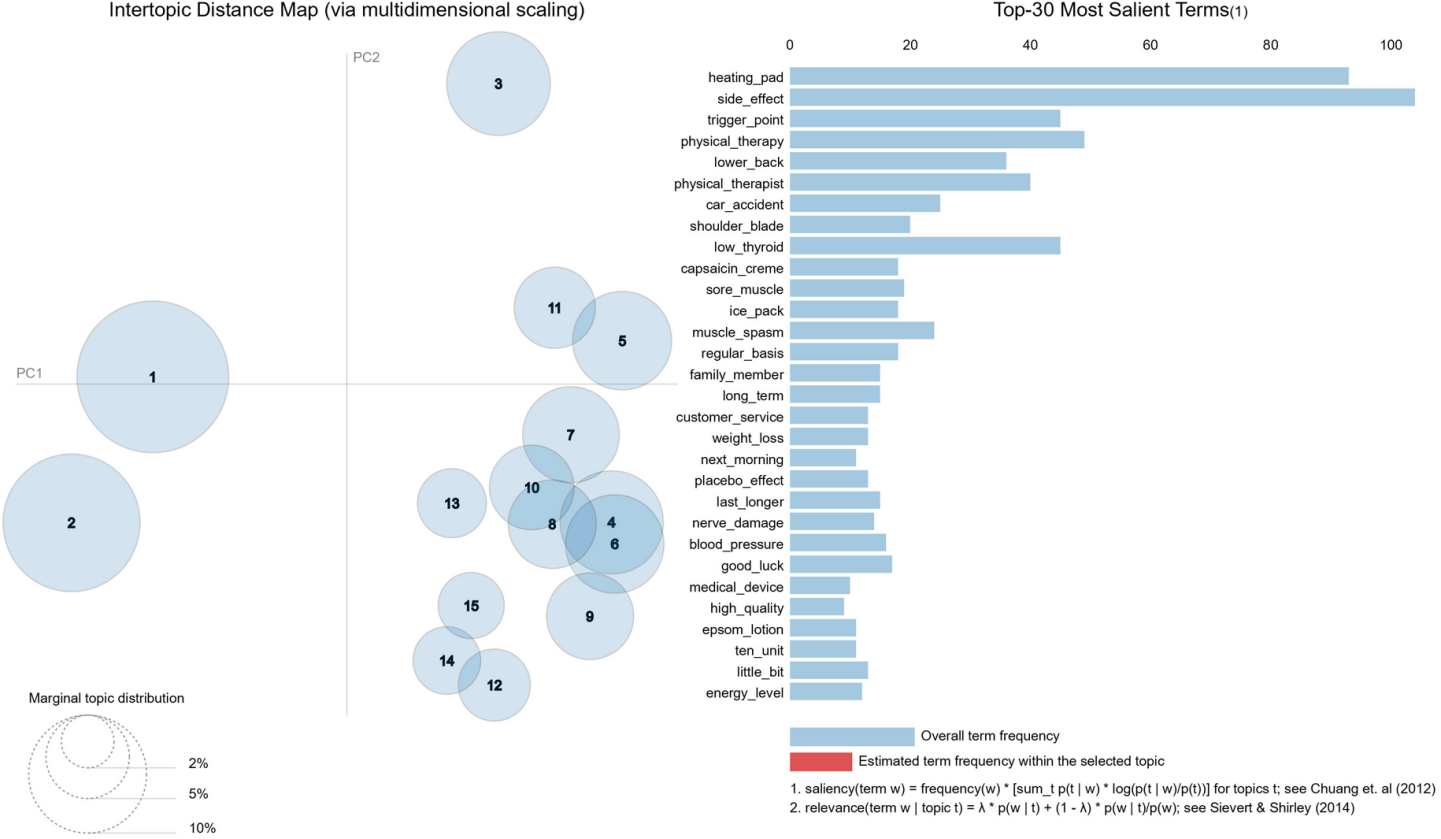
*LDAviz* result of the 15 topics with an inter-topic distance map and the top 30 most salient terms of the entire corpus. The circles in the left panel represent the sizes (prevalence) of the topics and their relative semantic distance from each other. The saliency bar chart in the right panel simply corresponds to term frequency in the corpus. The relative salient terms of each topic will update in the right panel upon clicking the specific circle on the left (the interactive feature is not presented here).

According to the peak cluster coherence score, the optimal number of topics was determined to be 15 on this dataset. The spatial rendering of the topics by *LDAviz* is shown in Figure 7, and the top five representative terms (bigrams only) for each topic are provided in Table 3. The identified topics do not appear to be clear-cut, especially considering the terms in each topic that diversely relate to pain conditions, product attributes, affected anatomy, and sentiment expressions. For example, topic #5 appears to involve both heating pads and deep tissue massage products, as well as their efficacy and affordability. A relatively clean topic is #11, which consists of positive reviews about valerian root, most likely for its use as a muscle relaxant.

**Table 3:** Top five salient terms of each LDA-discovered topic from the chronic pain-related product reviews.

Topic ID	Top five bigrams identified by the *LDAvis* program
1	Hydrogen peroxide, adrenal fatigue, breast cancer, stopthethyroidmadness website, prostate cancer
2	Low thyroid, lab test, better eye, macular degeneration, retinol palmitate
3	Side effect, muscle spasm, temporary relief, flat compress, touchstone
4	Physical therapy, physical therapist, ice pack, last longer, daily basis
5	Heating pad, immediate relief, much better, best price, deep tissue
6	Capsaicin creme, great deal, red tube, hyaluronic acid, white tube
7	Trigger point, blood pressure, prescription drug, sleep aid, recommended dosage
8	Car accident, shoulder blade, long term, little bit, good luck
9	Customer service, weight loss, Epsom lotion, 10 units, Epsom salt
10	Lower back, sore muscle, regular basis, knee replacement, degenerative disc
11	Valerian root, long period, muscle relaxant, big deal, positive review
12	Family member, placebo effect, nerve damage, instant relief, energy level
13	Dry mouth, plantar fasciitis, bulge disc, sports injury, minor ache
14	Next morning, high quality, medical device, work wonder, fatigue syndrome
15	Left knee, different types, larger size, honest review, many different

We used only bigrams in the topic modeling. Note that the program did not assign a single aggregated label for each topic in addition to these salient terms.

## 4. Discussion

### 4.1. Comments on the key findings

By searching the exact term “chronic pain,” we retrieved a corpus of 1,589 product reviews on Amazon.com within the category Health & Personal Care from 2001 to 2014. The discovered properties of the reviews align with our intuitive understanding. Most of the consumers (94% = 1,382/1,466) in the cohort posted only one review, with a median length of 107 words. Usual consumers apparently do not spend extensive effort writing product reviews all the time. The dominant readability level around the 9th–12th grade indicates the review language to be comprehensible for people with a high school education. Reviews of extreme length or difficulty appear to include excerpted third-party content such as scientific articles, e.g., when the consumer wanted to quote evidence to support an opinion.

The summarized product categories, such as pain medications and massage equipment, mostly appear to be familiar. Interestingly, there are fewer well-known but literature-supported items like magnesium (18). The rating distributions in Table 2 concur well with the J-shape phenomenon reported in previous research on Amazon product reviews (19). It suggested that the higher percentage of 4–5 star rating was driven by two biases: People who purchased a product were more likely to write positive reviews, and people with moderate views were less passionate about reporting their ratings (compared to those who wanted to “brag or complain”). The complaints made a noticeable uptick in 1-star ratings, although the counts are not comparable to the 4–5 stars in magnitude. Therefore, the dominant positive reviews should not be interpreted naively as indicating the actual population with CP was generally satisfied with these products.

In the analysis of Figure 6, some of the rating-differential features are intuitive, such as “highly recommend” and “great product”, for the positive rating. There are also ambiguous bigrams, including “chronic pain” itself, that hardly infer the sentiments without inspecting the context. For example, “years ago” is linked to many positive reviews where the consumer referred to the medical history of their chronic pain, which could be a common style of persuasion by offering personal stories in the review. The feature “pain relief” reads literally positive but is associated with negative reviews; by inspecting the wider contexts, it seems that the term often links to a failed fulfillment of what the product was supposed to achieve. This also indicates that the bigrams had limitations in adequately representing the sentiment. It is interesting that “heating pad” products specifically link with negative reviews. We inspected the contents and found that consumers frequently reported about quality issues and glitches in heating pad products.

The analyses of the other disorders mentioned in the reviews were encouraging. The network in Figure 5 shows information consistent with the literature on CP. For example, the etiology hubs such as fibromyalgia, cancer, and osteoarthritis all rank high as frequently co-occurring disorders with CP in PubMed. A notable discrepancy is back pain, which ranks at the top in Table 1 as the most frequently occurring concept with CP in PubMed, but it does not appear to stand out as the largest hub in Figure 5. Likewise, arthritis, as the second largest hub, does not have a direct presence in Table 1, but it is unclear how many of those occurrences refer to osteoarthritis (ranked 4th in Table 1). That said, there is no reason to believe that consumer activities must align perfectly with the dominant interest of the research community, and any such discrepancies could also lead to new research questions. For example, anxiety as a salient node in Figure 5 was not found to co-occur with chronic pain in Table 1, suggesting that some frequently observed comorbidities might have received limited attention from CP researchers.

The identified topics in Table 3 exhibit a mix of possible CP causes, side effects of the product (or a compared product), and many discursive concepts. Some of the terms require additional knowledge to see their relevance with CP. For example, topics #1 and #2 specifically discuss CP issues related to hypothyroidism (20), which might distinguish them from the other topics in Figure 7 along the first principal component axis (PC1). Although literature verification helped reveal some of the underlying associations, the topic modeling was suboptimal due to the pervasive loosely linked concepts. As a general caveat, consumer reviews still contain substantial noise (4, 21), so the results are better interpreted with reservation, given that we did not perform any auditing of possibly fake reviews. We chose to use only bigrams in the topic modeling after preliminary experiments showed noisy results from unigrams and trigrams. However, future experiments should consider using a mixture of *n*-grams as the input to see if meaningful variable-length terms could be prioritized by LDA.

### 4.2. Limitations

Notable limitations of the study include the following: The exact phrase “chronic pain” could miss lexical variants that also qualify pertinent cases. As an exploratory study, we did not perform exhaustive comparisons or optimizations when applying the individual methods. For example, we chose the Dale–Chall score based on the literature but did not compare it with other alternative readability measures. We leave the comparison and verification to future work. The Amazon product categories might not be rigorously curated and could moderately shift over time. The used dataset only covered up to 2014, which may not reflect the latest trends, especially regarding the products. We believe that the common health issues in the CP population are stable over time, but the verification requires future research. Without a reliable way to automatically identify all the CP-co-occurring MeSH concepts in the review text, we could not perform an extensive assessment of the coverage of literature-reported associations in the product reviews. This can be a fruitful direction to pursue in the future.

### 4.3. Conclusion and future work

We performed descriptive analyses for a set of health product reviews on CP. The majority of the reviews were concise and readable for high school-level English. The comorbidity network analysis suggests the cohort to be clinically representative of the health issues in people with CP but with a noted bias toward the “positive responders.” Despite the intrinsic noise in the product reviews, they might serve as a unique real-world data source for CP research. Overall, the results suggest that online consumer reviews contain rich information about the pain conditions and comorbidities experienced by CP patients, the types of OTC treatments they frequently sought, and their feedback from using the products.

Potential applications could include identifying gaps in existing therapies or candidates for clinical trials (i.e., to engage subjects with specific conditions and varying responses to the OTC products), yet the design and feasibility check are beyond the scope of this study. Likewise, we leave the exploration of additional mining techniques to future work, which should also address debiasing and cross-validating with a different data source, such as clinical trial results. Additional validation studies should compare the findings by expanding the cohort inclusion terms beyond “chronic pain” and potentially including the cohort's reviews on other relevant health products around the same period.

## Data Availability

The original contributions presented in the study are included in the article/**Supplementary Material**; further inquiries can be directed to the corresponding author.

## References

[1] Duenas M Ojeda B Salazar A Mico JA Failde I. A review of chronic pain impact on patients, their social environment and the health care system. J Pain Res. (2016) 9:457–67. 10.2147/JPR.S105892 27418853 PMC4935027

[2] Dahlhamer J Lucas J Zelaya C Nahin R Mackey S DeBar L Prevalence of chronic pain and high-impact chronic pain among adults – United States, 2016. MMWR Morb Mortal Wkly Rep. (2018) 67(36):1001–6. 10.15585/mmwr.mm6736a2 30212442 PMC6146950

[3] Doughman E. U.S. painkiller market to reach $5.9 billion by 2023: PharmaceuticalProcessingWorld.com (2019). Available at: https://www.pharmaceuticalprocessingworld.com/u-s-painkiller-market-to-reach-5-9-billion-by-2023/

[4] de Barra M. Reporting bias inflates the reputation of medical treatments: a comparison of outcomes in clinical trials and online product reviews. Soc Sci Med. (2017) 177:248–55. 10.1016/j.socscimed.2017.01.033 28190628

[5] Maharana A Cai K Hellerstein J Hswen Y Munsell M Staneva V Detecting reports of unsafe foods in consumer product reviews. JAMIA Open. (2019) 2(3):330–8. 10.1093/jamiaopen/ooz030 31984365 PMC6951857

[6] Torii M Tilak SS Doan S Zisook DS Fan JW. Mining health-related issues in consumer product reviews by using scalable text analytics. Biomed Inform Insights. (2016) 8(Suppl 1):1–11. 10.4137/BII.S37791 27375358 PMC4915789

[7] He R McAuley J. Ups and downs: modeling the visual evolution of fashion trends with one-class collaborative filtering. Proceedings of the 25th international conference on world wide web; Montréal, Québec, Canada: international world wide web conferences steering committee (2016). p. 507–17

[8] Qi P Zhang Y Zhang Y Bolton J Manning CD. Stanza: a python natural language processing toolkit for many human languages (2020) [arXiv:2003.07082 p.]. Available at: https://ui.adsabs.harvard.edu/abs/2020arXiv200307082Q

[9] Dale E Chall JS. A formula for predicting readability. Educ Res Bull. (1948) 27(1):11–28.

[10] Zhang Y Zhang Y Qi P Manning CD Langlotz CP. Biomedical and clinical English model packages for the stanza python NLP library. J Am Med Inform Assoc. (2021) 28(9):1892–9. 10.1093/jamia/ocab090 34157094 PMC8363782

[11] Dogan RI Leaman R Lu Z. NCBI disease corpus: a resource for disease name recognition and concept normalization. J Biomed Inform. (2014) 47:1–10. 10.1016/j.jbi.2013.12.006 24393765 PMC3951655

[12] Bastian M Heymann S Jacomy M. Gephi: an open source software for exploring and manipulating networks. Proc Int AAAI Conf Weblogs Soc Media. (2009) 3(1):361–2. 10.1609/icwsm.v3i1.13937

[13] National Library of Medicine. MEDLINE Co-Occurrences (MRCOC) files (2022). Available at: https://lhncbc.nlm.nih.gov/ii/information/MRCOC.html (Accessed April 26, 2022).

[14] Blondel VD Guillaume J-L Lambiotte R Lefebvre E. Fast unfolding of communities in large networks. J Stat Mech: Theory Exp. (2008) 2008(10):P10008. 10.1088/1742-5468/2008/10/P10008

[15] Blei DM Ng AY Jordan MI. Latent dirichlet allocation. J Mach Learn Res. (2003) 3:993–1022. 10.5555/944919.944937

[16] Sievert C Shirley K. LDAvis: A Method for Visualizing and Interpreting Topics. Baltimore, MD, United States: Association for Computational Linguistics (2014).

[17] Lundberg SM Lee S-I. A unified approach to interpreting model predictions. (2017). p. 4765–74

[18] Shin HJ Na HS Do SH. Magnesium and pain. Nutrients. (2020) 12(8):2184. 10.3390/nu12082184 32718032 PMC7468697

[19] Hu N Zhang J Pavlou PA. Overcoming the J-shaped distribution of product reviews. Commun ACM. (2009) 52(10):144–7. 10.1145/1562764.1562800

[20] Aloisi AM Vodo S Buonocore M. Pain and thyroid hormones. Neurol Sci. (2013) 34(9):1501–8. 10.1007/s10072-013-1440-7 23609461

[21] He S Hollenbeck B Proserpio D. The market for fake reviews. Mark Sci. (2022) 41(5):896–921. 10.1287/mksc.2022.1353

