# Supplementary material for: Retrospective content analysis of consumer product reviews related to chronic pain
Source: Front Digit Health. 2023 Apr 24;5:958338. doi: 10.3389/fdgth.2023.958338 (PMC10165495; doi:10.3389/fdgth.2023.958338)
Supplement: Supplementary file 3 [file Datasheet3.pdf]

Description of the review metadata

Data fields:

- asin - ID of the product, e.g. 0000031852
- title - name of the product
- price - price in US dollars (at time of crawl)
- imUrl - url of the product image
- related - related products (also bought, also viewed, bought together, buy after viewing)
- salesRank - sales rank information
- brand - brand name
- categories - list of categories the product belongs to

Example:

{

'asin': 'B00008WFLP',

'related': {

'also_bought': ['B001CP7B90', 'B001MSCW50', 'B00E4MLRY8', 'B001V9DJKW', 'B004LTEMXW'],

'also_viewed': ['B001CP7B90', 'B001MSCW50', 'B004LTEMXW', 'B00E4MLRY8', 'B000WZOJCI'],

'bought_together': ['B001CP7B90']

},

'title': 'Mobisyl Pain Relieving Creme with Soothing Aloe Vera, 8.0-Ounce Jar (Pack of 2)',

'price': 26.35,

'salesRank': {'Health & Personal Care': 30869},

'imUrl': 'http://ecx.images-amazon.com/images/I/51FuPj0CJUL._SX300_.jpg',

'brand': 'Mobisyl',

'categories': [

['Health & Personal Care', 'Health Care', 'Pain Relievers', 'Joint & Muscle Pain Relief', 'Treatments', 'Pain Relief Rubs']

],

'description': 'Penetrate to the site of muscle and joint pain with Mobisyl Crème. The special analgesic in Mobisyl penetrates deep through tissue to deliver relief directly to painful muscles and joints. Unlike other topical pain medications, Mobisyl does not burn or smell and

can be used under clothing or bandages. Apply Mobisyl to aching muscles or take a hot bath or shower, then rub in Mobisyl Crème to head off arthritis pain.'

}
