# Supplementary material for: Retrospective content analysis of consumer product reviews related to chronic pain
Source: Front Digit Health. 2023 Apr 24;5:958338. doi: 10.3389/fdgth.2023.958338 (PMC10165495; doi:10.3389/fdgth.2023.958338)
Supplement: Supplementary file 4 [file Datasheet4.pdf]

All features used in the logistic regression and SHapley Additive exPlanations (SHAP) analysis

10 minutes

10 years

15 minutes

15 years

20 minutes

20 years

30 minutes

aches pains

anti inflammatory

arthritis pain

best product

blood pressure

car accident

chronic fatigue

chronic pain

chronic pain condition

chronic pain fibromyalgia

chronic pain issues

chronic pain like

chronic pain pain

chronic pain sufferer

chronic pain ve

chronic pain years

chronic pains

constant pain

couple hours

day pain

decided try

deep tissue

definitely recommend

disc disease

does help

does work

doesn work

don know

don use

easy use

excellent product

fall asleep

feel better

feel like

felt like

fibromyalgia chronic

good luck

great deal

great product

heating pad

heating pads

help chronic

help chronic pain

highly recommend

highly recommend product

highly recommended

joint pain

just days

just like

knee pain

live chronic

live chronic pain

long term

long time

lot pain

love product

lower pain

make sure

months ago

muscle pain

muscle spasms

neck pain

neck shoulders

need use

nerve pain

night sleep

pain arthritis

pain chronic

pain completely

pain condition

pain day

pain does

pain fibromyalgia

pain free

pain gone

pain highly

pain inflammation

pain issues

pain just

pain killers

pain knee

pain left

pain level

pain like

pain management

pain medication

pain meds

pain muscle

pain neck

pain need

pain pain

pain really

pain relief

pain reliever

pain shoulder

pain sufferer

pain sufferers

pain swelling

pain used

pain ve

pain works

pain years

past years

people chronic

people chronic pain

physical therapist

physical therapy

plantar fasciitis

product chronic

product chronic pain

product does

quality life

read reviews

really help

really helped

really helps

really works

recommend chronic

recommend chronic pain

recommend product

relief chronic

relief chronic pain

relief pain

relieve pain

severe chronic

severe chronic pain

severe pain

shoulder pain

sleep night

sore muscles

started taking

started using

suffer chronic

suffer chronic pain

suffered chronic

suffered chronic pain

suffering chronic

suffering chronic pain

suffers chronic

suffers chronic pain

tens unit

times day

trigger point

twice day

use product

using product

ve taking

ve tried

ve used

ve using

weeks ago

weeks pain

work better

works better

works great

worth try

year old

years ago

years old

years ve
