# Supplementary material for: Retrospective content analysis of consumer product reviews related to chronic pain
Source: Front Digit Health. 2023 Apr 24;5:958338. doi: 10.3389/fdgth.2023.958338 (PMC10165495; doi:10.3389/fdgth.2023.958338)
Supplement: Supplementary file 5 [file Datasheet5.pdf]

Top 10 features that supported negative and positive review ratings

**Pro-negative**	**Pro-positive**
chronic pain	highly recommend
heating pad	years ago
heating pads	ve tried**
pain issues	great product
pain relief*	recommend product
physical therapy	easy use
help chronic*	joint pain
chronic pain issues	suffer chronic
help chronic pain*	quality life
relieve pain*	really helps

*The pro-negative features that appear to be positive by itself mostly occur in contexts of negative sentiment, e.g., “did not help chronic pain”.

**The “ve” resulted from the tokenizer behavior that broke “I’ve” into two words.
